# The Pathology of Carcinoma*Introducing a discussion on Carcinoma at a meeting of the New York State Medical Society, February 8, 1893.

**Published:** 1893-03

**Authors:** H. C. Coe

**Affiliations:** New York


### THE PATHOLOGY OF CARCINOMA.*

ABSTRACT OF PAPER BY DR. H. C. COE, NEW YORK.

Of all subjects in pathology there is none that has proved of such absorbing interest during the past five years as that of carcinoma. The great advances made in the studies of bacteriology have awakened the belief that by its aid the mystery surrounding this disease would finally be dispelled, and that some light would be gained as to its curative treatment.

The practical value of laboratory investigations has finally been admitted by surgeons, who have seen their results in the development of modern asepsis; it is to pathologists, then, that they must look for hints as to the radical cure of cancer, if such a cure is possible.

Virchow, five years ago, said that from a diagnostic standpoint it made no particular difference whether cancer develops from pre-existing epitheline or from the tissue of the deeper layers. The most divergent views have been advanced regarding its nature, some holding that it was the result of a retrograde metamorphosis in which the cell returns to its embryonal condition, but unlike the normal embryonal cells, that of cancer is not directly under the influence of the nervous system. Others have affirmed that the cells of cancer are functionless, but are endowed with an increased power of assimilation and proliferation. There is no reason to believe that cancer is of inflammatory origin. The disease is in the cell, which is essentially infectious, and tend to invade surrounding tissues, like leucocytes or bacteria. Whether this infectious quality is due to the presence of a parasitic element or not is still disputed, though there is strong evidence in favor of the former view. Furthermore, it is probable that the cancer-cell is itself primarily infected by coccydia which gain access to it from the surface of the body.

Metastasis is readily explained by the infection of epithetial

* Introducing a discussion on Carcinoma at a meeting of the New York State Medical Society, February 8, 1893.

cell in localities distant from the parent growth by cells carried from it through the lymphatics. Metastasis must be carefully distinguished from direct extension, when tissue adjacent to the neoplasm appears microscopically to be healthy is invaded by outlying groups of cancer-cells. This explains the rapid recurrence seen after amputation of the breast when the incision has not been carried sufficient far beyond the infected area.

The expression "cancerous cachexia" or "predisposition" is incorrect, since the cachexia observed in advanced carcinoma is merely the general depreciation of the system observed in all wasting diseases, to which is often added septic infection from the ulcerated area. Heredity is now regarded as only one in a series of etiological factors. Even persistent local irritation is not in itself enough to produce cancer, since there must be present also certain degenerative changes in the epithelial cells at the point of irritation. The same law applies to the cancerous metamorphosis of benignant neoplasms.

Inflammatory changes within a cancerous tumor are destructive; inflammation of the surrounding tissue may be to some extent conservative by destroying, or arresting the growth of infiltrating groups of cancer cells. Cancer may change its type, or even appear to become quiescent, as seen in epithelioma of the cervix uteri after cauterization. This may be due to impaired nutrition of the growth.

The practical lessons to be derived from the study of the pathology of cancer relate to prophylaxis and treatment. General or systemic prophylaxis is purely theoretical. Local prophylaxis consists in the careful observation of every suspicious area of tissue which is subject to continued irritation and its thorough removal, if possible, in the pre-cancerous stage, as in the case of the eroded cervix uteri.

The medicinal treatment of the disease has thus far yielded unsatisfactory results. There exists a general belief among the laity, shared by many of the profession, that a specific has been, or will be, discovered. Such a discovery is not beyond the the range of possibility, especially when we consider the earnest efforts which are now being made to discover the true nature of the disease. The indications for surgical treatment, based on pathological investigations are clear—to remove the neoplasm at the earliest possible stage, and to remove it thoroughly, sacrificing freely adjacent tissues that are apparently healthy, with the view of removing all outlying groups of cells. The surgeon should aim at thorough excision more than at rapid healing and a beautiful wound. In fact, inflammation may be a durable process rather than one to be sedulously avoided, since it may really be conservative, as before stated. In cancer of the breast, for example, no matter how limited, the axillary glands and fat should be thoroughly removed, even when they are not visibly affected. The use of the galvano cautery, so ably advocated by Byrne, for the removal of epithelioma of the cervix uteri, is based on a sound principle—the destructive action of the heat on ineffected tissue far beyond the visible and palpable area of infiltration.

